# Editorial

**Published:** 1864-08

**Authors:** 


C. S. Medical & Surgical Journal.

RICHMOND, AUGUST, 1864.

*AYRES & WADE......PUBLISHERS AND PROPRIETORS.*

EDITORIAL.

*External Application of Turpentine as a substitute for the internal use of Quinine in the treatment of Intermittent Fever.*

Since the publication of the Reports on the Treatment of Intermittent Fever, by the external use of the oil of turpentine, (*vide* January No. Art. 3,) seventy returns, involving over four hundred cases, have been received from different hospitals and posts, owing to instructions received from the Surgeon-General's office. With few exceptions, the remedy is regarded as one of great power, if not positive efficiency, in preventing a return of paroxysm. But, weighing the elements of these reports, candor compels us to say that our conclusions are not so positive.

We will, for a moment, consider the difficulties in determining such a proposition. When we take into consideration the difference between *intercurrent* and *recurrent* chill, it is obvious that experimental treatment will be very likely to mislead us. A patient, suffering from debility or a debilitating disease, may have a chill induced by climacteric, or even mental and moral influences; with proper care and freedom from exposure to these causes, the paroxysm will not be repeated, and certainly turpentine, if used, could claim no specialty under such circumstances. Even in recurrent chill, where the paroxysms occur periodically, it is impossible to determine the hour or the day of recurrence, for the quotidian may pass spontaneously into the tertian or quartan without any treatment at all. Therefore, if we attempt to meet the quotidian type with quotidian treatment, it may have already reverted to another type—even to the quartan; and we jubilate for two days over the value of a remedy that really has no result.

We know, moreover, that any powerful revulsion, whether mental, moral or physical, will prevent a chill, and over them turpentine has no special advantage, it only being one of that class. A blister in action, alcoholic stimulants, narcotic medicines, sudden shock, as from a plunge into cold water; exciting news, good or bad, stave off chills, and we all know this, but rarely resort to them therapeutically. In this view of the subject we may regard the labors of our reporters as useful, in calling our attention to the value of such revulsive agents.

Again, many persons have chill as it were constitutionally, that is, all their physical and intellectual disorders are characterized by this symptom. These cases will be cured by purgatives, by tonics, or nervines, but none of them by the antiperiodic treatment, *per se*. Some get well without any treatment, simply wearing out the influence; and some never get well, no matter what the treatment, until they have changed either their habits or their location.

In the cases before us, while a large number are reported as cured by this remedy *alone*, it does not weaken our proposition that many cases (especially intercurrent chill) get well without any treatment at all; and, in the bulk of the cases, the turpentine application was either preceded by purgatives, simple or mercurial, or accompanied by remedies of known tonic, antiperiodic, alterative, or nervine properties, thus introducing new complications which prevent a determination of the question.

Again, these cases, generally speaking, being intermittent fever of a mild type, the patients were often reported to duty, if in two or three days the chill fails to appear. Here is another grave impediment to frustrate our efforts after truth, for nothing is so common as that a chill will cease temporarily, except the certainty of its early recurrence.

We may conclude, therefore, that the terebinthinate externally applied, is one of the large class of agents which may be rendered useful in the treatment of periodic fever, as an adjuvant to other remedies, but that it does not deserve to be regarded as a specific in the treatment of such affections

